# Supplementary material for: Modeling glioblastoma heterogeneity as a dynamic network of cell states
Source: Mol Syst Biol. 2021 Sep 16;17(9):e10105. doi: 10.15252/msb.202010105 (PMC8444284; doi:10.15252/msb.202010105)
Supplement: Supplementary file 5 — Source Data for Figure 3 [file MSB-17-e10105-s001.zip › Figure3A_sourcedata/GSEA_3065/nosigdb_state4.GseaPreranked.1622718430994/LARSSON_STATE_6.html]

Details for gene set LARSSON\_STATE\_6[GSEA]

|  || Dataset | state4 |
| Phenotype | NoPhenotypeAvailable |
| Upregulated in class | na\_pos |
| GeneSet | LARSSON\_STATE\_6 |
| Enrichment Score (ES) | 0.98574334 |
| Normalized Enrichment Score (NES) | 4.200147 |
| Nominal p-value | 0.0 |
| FDR q-value | 0.0 |
| FWER p-Value | 0.0 |
Table: GSEA Results Summary

  

Fig 1: Enrichment plot: LARSSON\_STATE\_6      
 Profile of the Running ES Score & Positions of GeneSet Members on the Rank Ordered List

  

| PROBE | GENE SYMBOL | GENE\_TITLE | RANK IN GENE LIST | RANK METRIC SCORE | RUNNING ES | CORE ENRICHMENT || 1 | GFAP |  |  | 0 | 0.995 | 0.0283 | Yes |
| 2 | C1QL1 |  |  | 1 | 0.808 | 0.0512 | Yes |
| 3 | ID3 |  |  | 2 | 0.730 | 0.0719 | Yes |
| 4 | PMP2 |  |  | 3 | 0.727 | 0.0926 | Yes |
| 5 | SAT1 |  |  | 4 | 0.669 | 0.1116 | Yes |
| 6 | FABP7 |  |  | 5 | 0.656 | 0.1302 | Yes |
| 7 | S100B |  |  | 6 | 0.630 | 0.1481 | Yes |
| 8 | CEBPD |  |  | 7 | 0.521 | 0.1629 | Yes |
| 9 | FTH1 |  |  | 8 | 0.512 | 0.1775 | Yes |
| 10 | GABBR2 |  |  | 9 | 0.508 | 0.1919 | Yes |
| 11 | CRYAB |  |  | 10 | 0.494 | 0.2059 | Yes |
| 12 | BEX1 |  |  | 11 | 0.473 | 0.2194 | Yes |
| 13 | EEF1A1 |  |  | 12 | 0.471 | 0.2327 | Yes |
| 14 | FTL |  |  | 13 | 0.466 | 0.2460 | Yes |
| 15 | METRN |  |  | 14 | 0.462 | 0.2591 | Yes |
| 16 | TUBA1A |  |  | 15 | 0.460 | 0.2722 | Yes |
| 17 | NLRP1 |  |  | 16 | 0.425 | 0.2842 | Yes |
| 18 | SPARCL1 |  |  | 17 | 0.416 | 0.2960 | Yes |
| 19 | SOX4 |  |  | 18 | 0.402 | 0.3074 | Yes |
| 20 | MXD4 |  |  | 19 | 0.400 | 0.3188 | Yes |
| 21 | OLFM2 |  |  | 20 | 0.396 | 0.3300 | Yes |
| 22 | RAB31 |  |  | 21 | 0.388 | 0.3410 | Yes |
| 23 | TOMM7 |  |  | 22 | 0.380 | 0.3518 | Yes |
| 24 | FABP5 |  |  | 23 | 0.375 | 0.3625 | Yes |
| 25 | TUBB2B |  |  | 24 | 0.373 | 0.3731 | Yes |
| 26 | PTMS |  |  | 25 | 0.372 | 0.3836 | Yes |
| 27 | BRI3 |  |  | 26 | 0.369 | 0.3941 | Yes |
| 28 | MARCKS |  |  | 27 | 0.363 | 0.4044 | Yes |
| 29 | HOPX |  |  | 28 | 0.360 | 0.4146 | Yes |
| 30 | MAGED1 |  |  | 29 | 0.356 | 0.4248 | Yes |
| 31 | PNRC1 |  |  | 30 | 0.354 | 0.4348 | Yes |
| 32 | QKI |  |  | 31 | 0.345 | 0.4446 | Yes |
| 33 | SH3BGRL |  |  | 32 | 0.340 | 0.4543 | Yes |
| 34 | IRS2 |  |  | 33 | 0.338 | 0.4639 | Yes |
| 35 | ZMAT3 |  |  | 34 | 0.338 | 0.4735 | Yes |
| 36 | BEX3 |  |  | 35 | 0.337 | 0.4830 | Yes |
| 37 | COMMD6 |  |  | 36 | 0.334 | 0.4925 | Yes |
| 38 | OST4 |  |  | 37 | 0.325 | 0.5018 | Yes |
| 39 | MT3 |  |  | 38 | 0.318 | 0.5108 | Yes |
| 40 | HEY1 |  |  | 39 | 0.318 | 0.5198 | Yes |
| 41 | CTXN1 |  |  | 40 | 0.316 | 0.5288 | Yes |
| 42 | NEAT1 |  |  | 41 | 0.315 | 0.5377 | Yes |
| 43 | FEZ1 |  |  | 42 | 0.313 | 0.5466 | Yes |
| 44 | C4orf3 |  |  | 43 | 0.308 | 0.5554 | Yes |
| 45 | SERF2 |  |  | 44 | 0.307 | 0.5641 | Yes |
| 46 | C9orf16 |  |  | 45 | 0.306 | 0.5728 | Yes |
| 47 | PFN2 |  |  | 46 | 0.303 | 0.5814 | Yes |
| 48 | ATP6V1G1 |  |  | 47 | 0.303 | 0.5900 | Yes |
| 49 | SOX9 |  |  | 48 | 0.299 | 0.5984 | Yes |
| 50 | TSC22D4 |  |  | 49 | 0.298 | 0.6069 | Yes |
| 51 | GABARAPL2 |  |  | 50 | 0.297 | 0.6153 | Yes |
| 52 | SNX3 |  |  | 51 | 0.297 | 0.6238 | Yes |
| 53 | MARCKSL1 |  |  | 52 | 0.295 | 0.6321 | Yes |
| 54 | C1orf122 |  |  | 53 | 0.294 | 0.6405 | Yes |
| 55 | BNIP3L |  |  | 54 | 0.294 | 0.6488 | Yes |
| 56 | GNAI2 |  |  | 55 | 0.293 | 0.6572 | Yes |
| 57 | ID2 |  |  | 56 | 0.292 | 0.6655 | Yes |
| 58 | SEPT7 |  |  | 57 | 0.292 | 0.6738 | Yes |
| 59 | TPT1 |  |  | 58 | 0.292 | 0.6821 | Yes |
| 60 | C4orf48 |  |  | 59 | 0.291 | 0.6903 | Yes |
| 61 | EFTUD2 |  |  | 60 | 0.290 | 0.6985 | Yes |
| 62 | TUBB2A |  |  | 61 | 0.287 | 0.7067 | Yes |
| 63 | FHL1 |  |  | 62 | 0.286 | 0.7148 | Yes |
| 64 | PHPT1 |  |  | 63 | 0.282 | 0.7228 | Yes |
| 65 | CD74 |  |  | 64 | 0.281 | 0.7308 | Yes |
| 66 | RND2 |  |  | 65 | 0.281 | 0.7388 | Yes |
| 67 | DKK3 |  |  | 66 | 0.281 | 0.7468 | Yes |
| 68 | RTL8C |  |  | 67 | 0.280 | 0.7547 | Yes |
| 69 | SESN3 |  |  | 68 | 0.278 | 0.7626 | Yes |
| 70 | RPL34 |  |  | 69 | 0.278 | 0.7705 | Yes |
| 71 | COL4A3BP |  |  | 70 | 0.276 | 0.7784 | Yes |
| 72 | CTNNA1 |  |  | 71 | 0.274 | 0.7861 | Yes |
| 73 | FOXO3 |  |  | 72 | 0.273 | 0.7939 | Yes |
| 74 | FAM20C |  |  | 73 | 0.272 | 0.8016 | Yes |
| 75 | MARCH2 |  |  | 74 | 0.269 | 0.8093 | Yes |
| 76 | NFIB |  |  | 75 | 0.269 | 0.8169 | Yes |
| 77 | CCNI |  |  | 76 | 0.266 | 0.8245 | Yes |
| 78 | CITED1 |  |  | 77 | 0.265 | 0.8320 | Yes |
| 79 | PFDN5 |  |  | 78 | 0.265 | 0.8395 | Yes |
| 80 | ZNF428 |  |  | 79 | 0.264 | 0.8470 | Yes |
| 81 | RPS11 |  |  | 80 | 0.262 | 0.8545 | Yes |
| 82 | LAMTOR4 |  |  | 81 | 0.262 | 0.8619 | Yes |
| 83 | PLEKHB1 |  |  | 82 | 0.262 | 0.8693 | Yes |
| 84 | SCP2 |  |  | 83 | 0.261 | 0.8767 | Yes |
| 85 | ID1 |  |  | 84 | 0.261 | 0.8842 | Yes |
| 86 | TTC3 |  |  | 85 | 0.261 | 0.8916 | Yes |
| 87 | PBX1 |  |  | 86 | 0.260 | 0.8989 | Yes |
| 88 | FYN |  |  | 87 | 0.260 | 0.9063 | Yes |
| 89 | SMDT1 |  |  | 88 | 0.259 | 0.9137 | Yes |
| 90 | LGALS3 |  |  | 89 | 0.259 | 0.9210 | Yes |
| 91 | FKBP8 |  |  | 90 | 0.258 | 0.9283 | Yes |
| 92 | SKP1 |  |  | 91 | 0.257 | 0.9356 | Yes |
| 93 | TXNIP |  |  | 92 | 0.254 | 0.9429 | Yes |
| 94 | MAP1LC3A |  |  | 93 | 0.253 | 0.9501 | Yes |
| 95 | SRP14 |  |  | 94 | 0.252 | 0.9572 | Yes |
| 96 | CLU |  |  | 95 | 0.252 | 0.9644 | Yes |
| 97 | ATP6V1F |  |  | 96 | 0.251 | 0.9715 | Yes |
| 98 | TNFRSF21 |  |  | 97 | 0.251 | 0.9786 | Yes |
| 99 | RPS27 |  |  | 98 | 0.250 | 0.9857 | Yes |
| 100 | HNRNPDL |  |  | 6028 | -0.251 | 0.0308 | No |
| 101 | HNRNPA2B1 |  |  | 6029 | -0.251 | 0.0380 | No |
Table: GSEA details [plain text format]

  

Fig 2: LARSSON\_STATE\_6: Random ES distribution      
 Gene set null distribution of ES for **LARSSON\_STATE\_6**
